# Pangenomic and immunoinformatics based analysis of Nipah virus revealed CD4^+^ and CD8^+^ T-Cell epitopes as potential vaccine candidates

**DOI:** 10.3389/fphar.2023.1290436

**Published:** 2023-11-14

**Authors:** Syed Aun Muhammad, Jinlei Guo, Komal Noor, Aymen Mustafa, Anam Amjad, Baogang Bai

**Affiliations:** ^1^ Institute of Molecular Biology and Biotechnology, Bahauddin Zakariya University, Multan, Pakistan; ^2^ School of Intelligent Medical Engineering, Sanquan College of Xinxiang Medical University, Xinxiang, China; ^3^ University of Health Sciences Lahore, Lahore, Pakistan; ^4^ School of Information and Technology, Wenzhou Business College, Wenzhou, China; ^5^ Zhejiang Province Engineering Research Center of Intelligent Medicine, Wenzhou, China; ^6^ The 1st School of Medical, School of Information and Engineering, The 1st Affiliated Hospital of Wenzhou Medical University, Wenzhou, China

**Keywords:** Nipah virus, Pangenome analysis, reverse vaccinology, CD4 + and CD8 + T-cell epitopes, physicochemical properties

## Abstract

**Introduction:** Nipah (NiV) is the zoonotic deadly bat-borne virus that causes neurological and respiratory infections which ultimately lead to death. There are 706 infected cases reported up till now especially in Asia, out of which 409 patients died. There is no vaccine and effective treatment available for NiV infections and we have to timely design such strategies as world could not bear another pandemic situation.

**Methods:** In this study, we screened viral proteins of NiV strains based on pangenomics analysis, antigenicity, molecular weight, and sub-cellular localization. The immunoproteomics based approach was used to predict T-cell epitopes of MHC class-I and II as potential vaccine candidates. These epitopes are capable to activate CD4^+^, CD8^+^, and T-cell dependent B-lymphocytes.

**Results:** The two surface proteins including fusion glycoprotein (F) and attachment glycoprotein (G) are antigenic with molecular weights of 60 kDa and 67 kDa respectively. Three epitopes of F protein (VNYNSEGIA, PNFILVRNT, and IKMIPNVSN) were ranked and selected based on the binding affinity with MHC class-I, and 3 epitopes (VILNKRYYS, ILVRNTLIS, and VKLQETAEK) with MHC-II molecules. Similarly, for G protein, 3 epitopes each for MHC-I (GKYDKVMPY, ILKPKLISY, and KNKIWCISL) and MHC-II (LRNIEKGKY, FLIDRINWI, and FLLKNKIWC) with substantial binding energies were predicted. Based on the physicochemical properties, all these epitopes are non-toxic, hydrophilic, and stable.

**Conclusion:** Our vaccinomics and system-level investigation could help to trigger the host immune system to prevent NiV infection.

## 1 Introduction

Nipah is a fatal bat-borne virus that can infect both humans and animals. In 1998, it first appeared in Malaysia and then spread to India, Singapore, and Bangladesh (Shariff, 2019). Fruit bats, especially *Pteropus* species are the natural host of the NiV (Soman Pillai et al., 2020). In September 1998, the first outbreak of NiV took place in Malaysia (Aljofan, 2013). When the initial outbreak occurred in Bangladesh in April 2001, 13 cases of NiV were identified (Ang et al., 2018). The first epidemic case was reported in India in January and February 2001. There have been 706 infected patients recorded so far, with 409 patients died. According to genetic analysis, the NiV has two strains, i.e., NiV-M and NiV-B for Malaysian and Bangladesh strains respectively. These two strains were the sources of epidemics in different parts of the world (Harcourt et al., 2005). Since 2001, a total of 325 human NiV cases with a fatality rate of 71% were reported in Bangladesh (Agrawal et al., 2023). Recently, 11 cases of NiV including eight deaths with a case fatality rate of 73% were reported in Bangladesh between January 2023 to February 2023 (WHO, 2023a). The most recent outbreak of NiV occurred in Kerala state of India between 12 and 15 September where six cases were reported including two deaths (WHO, 2023b).

It is an enveloped non-segmented RNA virus with a genome size of roughly 18.2 kb that belongs to the *Paramyxoviridae* family and the genus *henipavirus*. The virus contains single-stranded RNA (ssRNA) (King et al., 2011) containing six genes. The proteins are encoded by these six genes including nucleocapsid (N), matrix protein (M), phosphoprotein (P), attachment fusion (F), attachment glycoprotein (G), and long polymerase (L) protein (Yahya et al., 2021). The order and pattern of all proteins is 3′N-P-M-F-G-L 5′ (Sun et al., 2018). NiV is made up of ribonucleoprotein (RNP) surrounded by a viral envelope containing L, N, and P proteins (Cox and Plemper, 2017).

**FIGURE 1:**
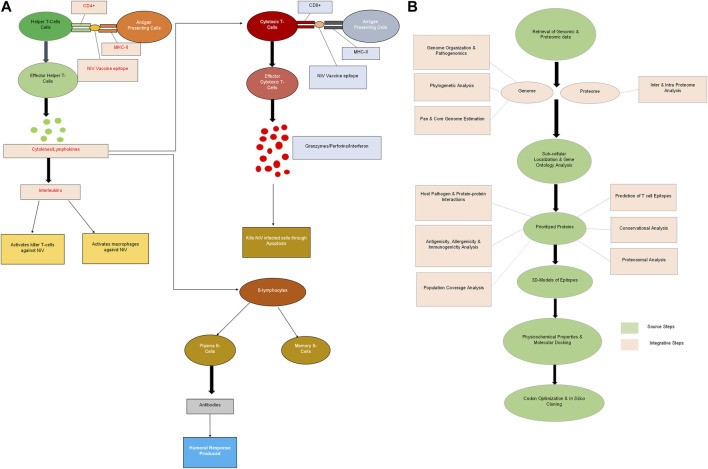
**(A)** Hypothetical model presenting the interaction of NiV epitopes with T-lymphocytes to activate host immune system **(B)** Integrative framework of our methodology to design potential NiV vaccine candidates.

The risk factors include intimate association with animal reservoirs, contaminated food consumption, and close association with animals infected with NiV (Ambat et al., 2019). The bat’s excretory products included urine, feces, and urine was the other common source of transmission (Hauser et al., 2021). Nosocomial infections, such as close contact with patients who were infected or touching infected surgical instruments were the additional risk factors. The infection period for the Malaysian epidemic lasted between 5 days and 2 months, while it took 10 days for the Bangladeshi outbreak to emerge. Indian isolates had an incubation period of 7–14 days. Vomiting, deterioration, headache, myalgia, behavioral changes, fever, pneumonia, and coughing were some of the typical symptoms. However, acute encephalitis and respiratory illness were the severe symptoms (Sharma et al., 2019). NiV diagnosis is a crucial step since NiV is a BSL-4 virus and is challenging to treat. The samples taken from human patients came from blood, cerebrospinal fluid (CSF), urine, throat swabs, and urine. Immunohistochemistry, virus isolation, histopathology, serological and molecular testing, and neutralization are all components of the diagnosing process. For its diagnosis, PCR is the most popular technique. Scientists have administered drugs including ribavirin, acyclovir, and favipiravir to affected individuals. Yet, no vaccination has been approved (Soman Pillai et al., 2020), so this study is significant to address this issue. We studied the genomic conservation across all strains to highlight the association of these strains and genomic differences (Sherman and Salzberg, 2020) and to design subunit vaccines based on T-cell epitopes to activate the immune system (Figure 1A). The potential vaccine candidates are designed using the reverse vaccinology (RA) and pangenomics. Due to the pathogen and antigens diversity, immunoinformatics greatly contributed to our understanding of the function of the immune system. This method of designing vaccines is relatively simple, economical, time-consuming, and accurate (Oli et al., 2020). The objective of this study is to design and identify the CD4^+^ and CD8^+^ T-cell epitopes of Nipah virus based on pangenomic and immunoinformatics system level analysis. We used integrative framework to evaluate the antigenicity, molecular modeling and interaction studies, simulation analysis and physicochemical predictions and structural stability analysis of potential NiV epitopes.

## 2 Materials and methods

### 2.1 Accession of data

The Virus Pathogen Resource Database (VIPR) was used to obtain the genomic and proteomic sequences (structural and functional genes) of NiV. The database includes complete genomic and proteomic information of each variant (Pickett et al., 2012). The integrative framework of our study (Figure 1B) was carried out using tools, software, online servers and databases (Table 1) to predict potential vaccine candidates of NiV.

**TABLE 1:** List of Tools/Databases/Servers used in the Research.

Sr. No.	Database	Uses	Web link	References
1	VIPR	Viral database	https://www.viprbrc.org/	Pickett et al. (2012)
2	Galaxy	Genome annotation	https://usegalaxy.org/	Jalili et al. (2020)
3	Roary	Pangenome analysis	https://sanger-pathogens.github.io/Roary/	Page et al. (2015)
4	Virus-mPLoc	Sub cellular localization	http://www.csbio.sjtu.edu.cn/cgi-bin/VirusmPLoc.cgi	Shen and Chou (2010)
5	VaxiJen	Proteins Antigenicity	http://www.ddg-pharmfac.net/vaxijen/VaxiJen/	Doytchinova and Flower (2007)
6	Propred	MHC-II epitope prediction	http://crdd.osdd.net/raghava/propred/	Singh and Raghava (2001)
7	IEDB	Immunogenicity prediction of MHC-I	http://tools.iedb.org/immunogenicity/	Calis et al. (2013)
8	Allergen FP	Allergenicity	https://ddg-pharmfac.net/AllergenFP/	Dimitrov et al. (2014)
9	ToxinPred	Toxicity prediction	http://crdd.osdd.net/raghava/	Gupta et al. (2013)
10	ProtParam	Physicochemical properties	https://web.expasy.org/	Gasteiger et al. (2005)
11	NetChop	Proteosomal Cleavage Analysis	http://tools.iedb.org/netchop/	Nielsen et al. (2005)
12	Pepfold	3D structure of epitopes	https://bioserv.rpbs.univ-paris-diderot.fr/services/PEP-FOLD/	Shen et al. (2014)
13	ERRAT	Model Quality estimation	https://saves.mbi.ucla.edu/	Colovos and Yeates (1993)
14	MINT	PPI analysis	https://mint.bio.uniroma2.it	Chatr-Aryamontri et al. (2007)
15	Cytoscape	PPI network	https://cytoscape.org/	Shannon et al. (2003)
16	JCAT	Codon Optimization	http://www.jcat.de/	Grote et al. (2005)

### 2.2 Multiple Sequence Alignment and phylogenetic analysis

Multiple Sequence Alignment (MSA) was performed to analyze the sequences of NiV variants to observe the differences and similarities among the isolates (Edgar and Batzoglou, 2006). Comparative assessment is important to determine structural and functional role of proteins. Proteins with less than 70% sequence similarity were excluded from further analysis. To analyze the evolutionary relationships of NiV variant, the phylogenetic tree was constructed using MEGA-11 software (Hassan et al., 2016) based on Neighbor-Joining method with 1000 bootstrap values.

### 2.3 Pangenomic analysis

The ROARY tool was used to carry out pan-genomic analysis (Page et al., 2015). Initially, Galaxy PROKKA (Prokaryotic Genome Annotation) tool was applied to annotate the entire genome of NiV isolates, transforming the initial genomic datasets into functional proteins (Jalili et al., 2020).

### 2.4 Subcellular localization and antigenicity prediction

The Virus-mPLoc tool was used to predict the subcellular localization elaborating the subcellular location of proteins within the host cell (Shen and Chou, 2010). The antigenicity determination is a crucial step as antigenic proteins can elicit an immune response. The VaxiJen server was used to predict the antigenicity of core proteins based on the threshold level set at 0.45 (Doytchinova and Flower, 2007). To analyze the molecular weight (m/w) of antigenic proteins, the ProtParam server (Gasteiger et al., 2005) was applied.

### 2.5 T-cell epitopes prediction and 3D modelling of epitopes

MHC class-I and II specific epitopes were predicted by ProPred tool (Singh and Raghava, 2001). These T-cell epitopes were further verified by the HLAPred tool (Adams and Koziol, 1995). To find the binding affinities of epitopes and asses how epitopes attach to the MHCs molecules as targets, 3D models of epitopes were constructed by the PEP-FOLD server (Shen et al., 2014; Du et al., 2021). The epitope sequence was positioned and labelled in a protein structure using the Chimera software (Goddard et al., 2018). The ERRAT tool was used to estimate the 3D structure’s quality (Colovos and Yeates, 1993). The amino acids sequences in favorable and unfavorable regions were determined by Ramachandran plots (Gopalakrishnan et al., 2007). The QMEAN server computed the qualitative Model Energy Analysis score of epitopes, and it was used to assess the similarity between predicted and experimental structure (Studer et al., 2020).

### 2.6 Identifying epitope physicochemical properties

We determined the physicochemical parameters of the epitopes. CamSol server was applied to analyze the solubility of epitopes that would help to determine how quickly it would dissolve in a solvent (Sormanni et al., 2017). ToxinPred, a server for the prediction of toxicity, charge, and SVM score were used for screening of epitopes (Gupta et al., 2013). Similarly, we calculated the half-life, aliphatic index, molecular weight, grand average hydropathicity (GRAVY), and instability index of all epitopes (Gasteiger et al., 2005) by using the ProtParam tool. We analyzed the allergenicity of epitopes by AllergenFp server (Dimitrov et al., 2014).

### 2.7 Conservational analysis and population coverage of epitopes

The conservation evaluation is important to study the conservancy of epitopes among all NiV variants using IEDB tool (Bui et al., 2007). The fraction of the epitope in human alleles was studied by the population coverage analysis using the same tool (Bui et al., 2006). To evaluate the spectrum and overall effect, we looked at the conservation of our selected epitopes among different variants of NiV. The degree of conservancy of an epitope within a given protein sequences was calculated using the Epitope Conservancy Analysis tool. BLAST analysis at NCBI server was performed against human proteome in order to exclude NiV epitope conservancy with human amino acid sequences.

### 2.8 Proteasomal cleavage and virus-host protein-protein interaction network analysis

Proteasomal cleavage is a necessary step for MHC-I epitopes because the process of breaking down proteins into small peptides that can be displayed on the MHC-I surface took place through this process. The NetChop tool was used for proteasomal cleavage analysis (Nielsen et al., 2005). The selected proteins were allowed to form a network to investigate how the proteins of NiV interact with human proteins. The Molecular Interaction Database identified the NiV proteins involved in interaction with human proteins (MINT) (Chatr-Aryamontri et al., 2007). The interactive network was built via Cytoscape server that connects the source protein to the target proteins (Shannon et al., 2003).

### 2.9 Binding energy and *in-silico* cloning

Using the Molecular Operating Environment (MOE) software, the binding affinity of the selective epitopes with MHCs molecules was analyzed. Ligands with low binding energies bind to the immune receptors more precisely (Tomar et al., 2010). The MHC-I and II as protein targets were docked with the 3D structure of epitopes. After the optimization of amino acid codon sequence, the monovalent vaccine construct is cloned. Codon optimization is a technique of enhancing the codon composition of a peptide without changing its sequence so that it may be expressed in a plasmid vector. Java Codon Adaptation Tool (JCAT) was applied for adapting the codons at default parameters, and the *E. coli* K12 strain was selected as the host organism. It provided the GC content and the value of the Codon Adaptation Index (CAI) (Grote et al., 2005). Codon optimization resulted the nucleotide sequence was used to clone the desired fragment. SnapGene tool was applied in cloning and it was carried out by the plasmid vector *E. coli* pET30a (+) and BamHI and HindIII enzymes were used for restriction.

### 2.10 Molecular dynamics simulations

The epitopes-MHCs complex was subjected to 100 ns of MD simulations with previous minimization and NVT/NPT equilibration phases. For the MD simulations, Amber99SB-ILDN protein force-field and TIP3P water model (Ochoa et al., 2022) were employed along with GROMACS v5.1. A minimum of 8 from each protein atom, the protein was solvated in a cubic water box with periodic boundaries. Na^+^ and Cl-counterions were added to the solvent after solvation to keep the box neutral. The Particle Mesh Ewald (PME) approach with 1.0 nm short-range electrostatic and van der Waals cutoffs was used to calculate the electrostatic interactions. To enable quick exploration of the conformational space, the simulations were run at a temperature of 350 K. To keep the system stable at this temperature, we employed a modified Berendsen thermostat (Ochoa et al., 2019) and a Parrinello-Rahman barostat (Okumura et al., 2007), and we constrained any receptor atoms that were more than 12 away from any peptide atom. The peptide’s flexibility was maintained, as were the receptor’s atoms that were closer to the threshold. By counting the number of hydrogen bonds between the peptide and protein, the number of heavy atom interactions, the all-atom root mean-square deviation (RMSD) of the peptide, and the root mean-square fluctuation of the protein and peptide, the simulations’ convergence was observed.

The MD trajectory conformations were scored using a variety of scoring systems. The majority of the scoring functions are statistical and knowledge-based potentials utilized for protein-protein and protein-ligand docking, however semiempirical techniques were also used. The total conformations of the protein-peptide complex were used to determine the scores. For each complex, the average score and standard deviation were determined using the MD trajectories from the last quarter (Guedes et al., 2018).

We determined whether the experimental activity difference (ΔΔ*G*) and the projected score difference for each potential pair of peptides coincide on the sign of the difference. Based on this, we determined whether a peptide, when compared to another, boosts or decreases the activity as a dichotomous response, individually for each scoring function and in a consensus framework. We checked the consensus strategies to see if the prediction matches the experimental ΔΔ*G* between peptides A and B (ΔΔ*G*_*AB*_). A linear regression model serves as the foundation of the first consensus strategy. In this instance, the response variable is the anticipated ΔΔ*G*, and the independent variables are the scores for each pair of peptides that differ from one another. When the experimental and anticipated ΔΔ*G* signs coincide, the prediction is said to be accurate. We used a leave-out-one training and testing method to cross-validate the model (Ochoa et al., 2019). One peptide was taken out of the training set, and the test group was constructed using any conceivable pairs that could be created between the taken-out peptide and the rest of the peptides. For every peptide that was accessible, the procedure was repeated. The final performance of all the sets produced using the leave-one-out technique was averaged in order to evaluate the resilience of the model. Regression of the sign of the ΔΔ*G*_*AB*_ for each pair of peptides A and B using logistic analysis is the second consensus technique. Where, the average score for peptide A is represented by *S*_*k*_^*A*^, and for peptide B by *S*_*k*_^*B*^. The bitstrings are shown:

$$\left\{\begin{array}{c} 1,sign\left(S_{k}^{A}-S_{k}^{B}\right)=sign\left(∆∆G_{AB}\right)\\ 0,otherwise \end{array}\right.$$

The linear regression model uses the same training and validation strategy (also known as the leave-one-out approach). In order to apply the bootstrapping method to evaluate the errors, we selected the consensus strategy with the best performance out of the two that we tested. To execute the consensus procedure, we repeatedly employed arbitrary blocks of the mean scores. The final accuracy was calculated by averaging all of the units, and it was then compared to the earlier consensus conclusions.

## 3 Results

### 3.1 Multiple Sequence Alignment and proteomic analysis

**FIGURE 2:**
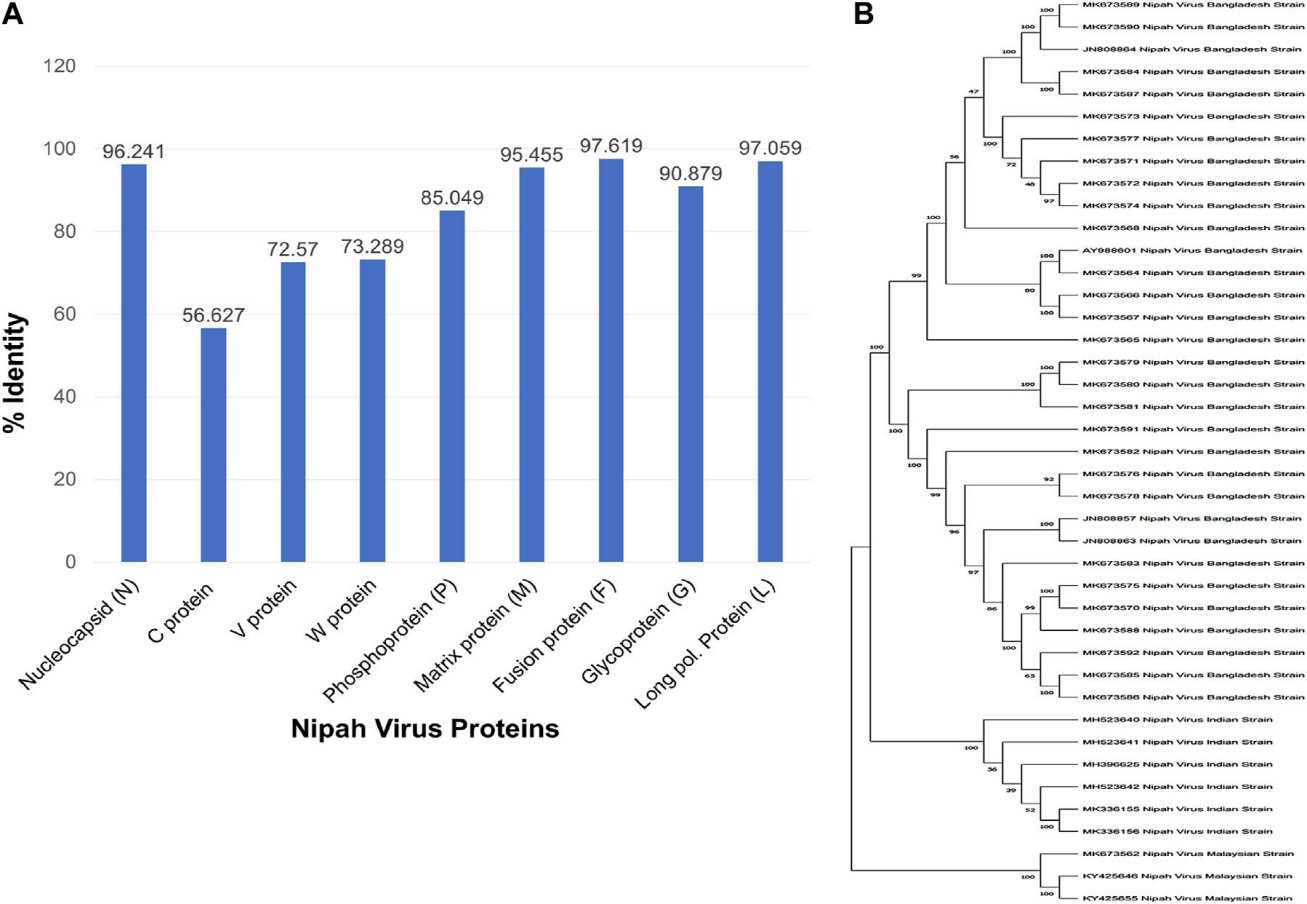
**(A)** Multiple sequence alignment and sequence similarity analysis of different proteins of NiV isolates **(B)** Evolutionary and phylogenetic tree of 1000 Bootstrap replicates of NiV isolates.

The FASTA sequences of nine proteins with 41 isolates were analyzed. Additionally, the full genomic sequences of all isolates were retrieved (Table 2). To determine the degree of similarity between isolates, each antigen was examined. The NiV “C” protein showed 56.6 percent similarity, indicating that it cannot be used as potential vaccine candidates while other proteins demonstrated greater than 70% similarity (Figure 2A).

**TABLE 2:** Pangenes of characterized isolates of Nipah Virus.

Sr. No.	Region	No. of isolates	Accession no.	Pangenes		
				Core genes	Shell genes	Cloud genes
1	Malaysia	3	MK673562, KY425655, KY425646	L G M N F	P/V/C group_1	group_7 group_8 group_9
2	Bangladesh	32	AY988601, JN808857, JN808863, JN808864, MK673564, MK673565, MK673566, MK673567, MK673568, MK673570, MK673571, MK673572, MK673573, MK673574, MK673575, MK673576, MK673577, MK673578, MK673579, MK673580, MK673581, MK673582, MK673583, MK673584, MK673585, MK673586, MK673587, MK673588, MK673589, MK673590, MK673591, MK673592			
3	India	6	MH396625, MH523642, MH523640, MH523641, MK336155, MK336156			

### 3.2 Phylogenetic and pan-genome analysis

**FIGURE 3:**
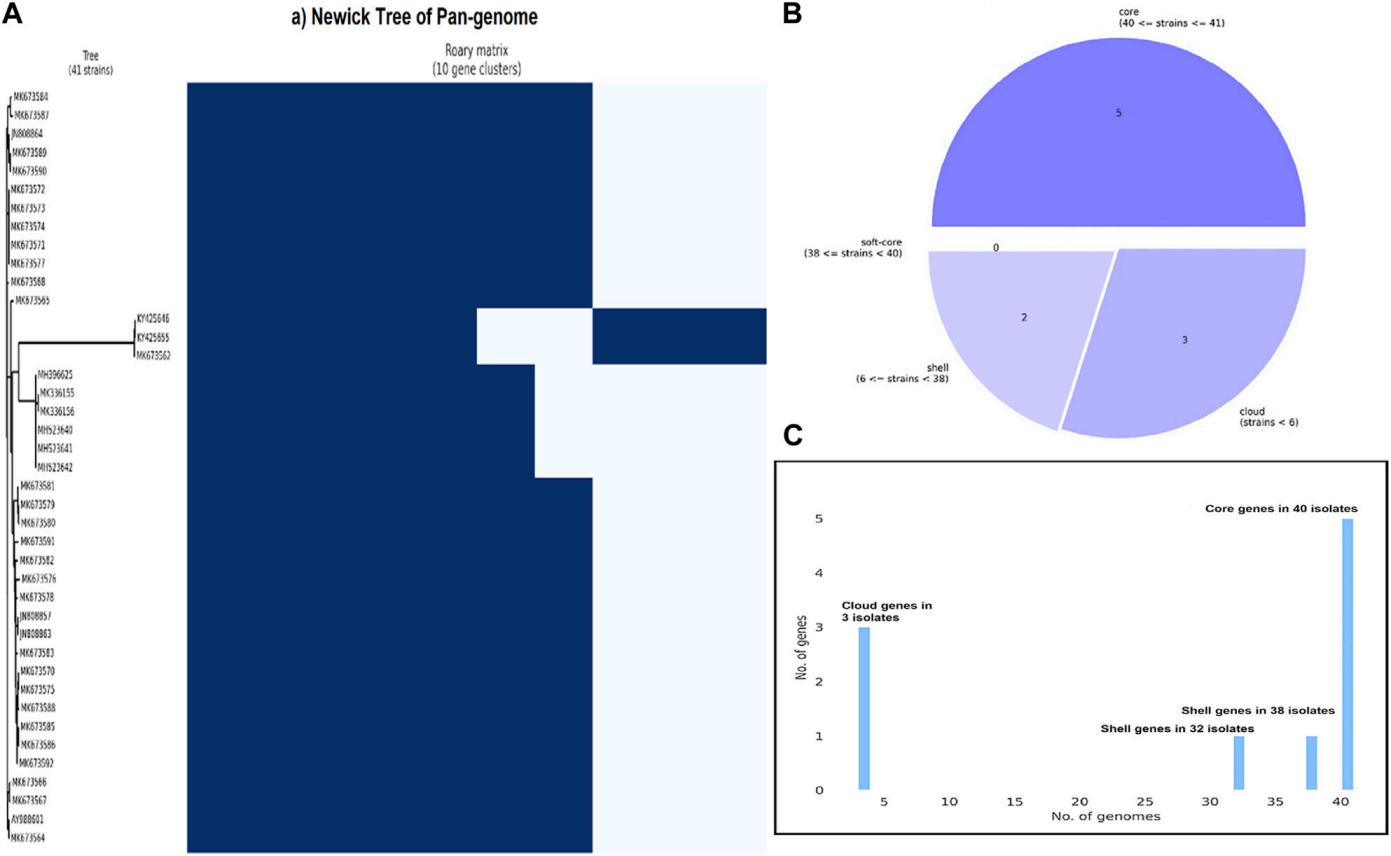
**(A)** Newick tree of 41 isolates with the conserved area highlighted in blue **(B)** Pie-chart displaying classification of core, soft-core, cloud, and shell genes in NiV isolates **(C)** Distribution and frequency of total number of genes NiV isolates.

All NiV isolates were evaluated by the phylogenetic tree. The distance demonstrated the evolutionary distance among various strains. The degree of divergence between isolates increases with increasing distance. Since the virus was originally propagated in Malaysia, the MK673562, KY425655, and KY425646 were the ancestors of all isolates (Figure 2B). The literature mining showed that the isolate AY988601 is more pathogenic compared to other variants. According to a pan-genome analysis, there are total number of 10 genes including 5 core genes, 2 shell genes, and 3 cloud genes, but there was no soft-core genes (Table 2). The F, M, N, G, and L proteins of NiV are among the core genes, which account for 95% of genome conservation (Figure 3).

### 3.3 Sub-cellular localization and antigenicity prediction

**FIGURE 4:**
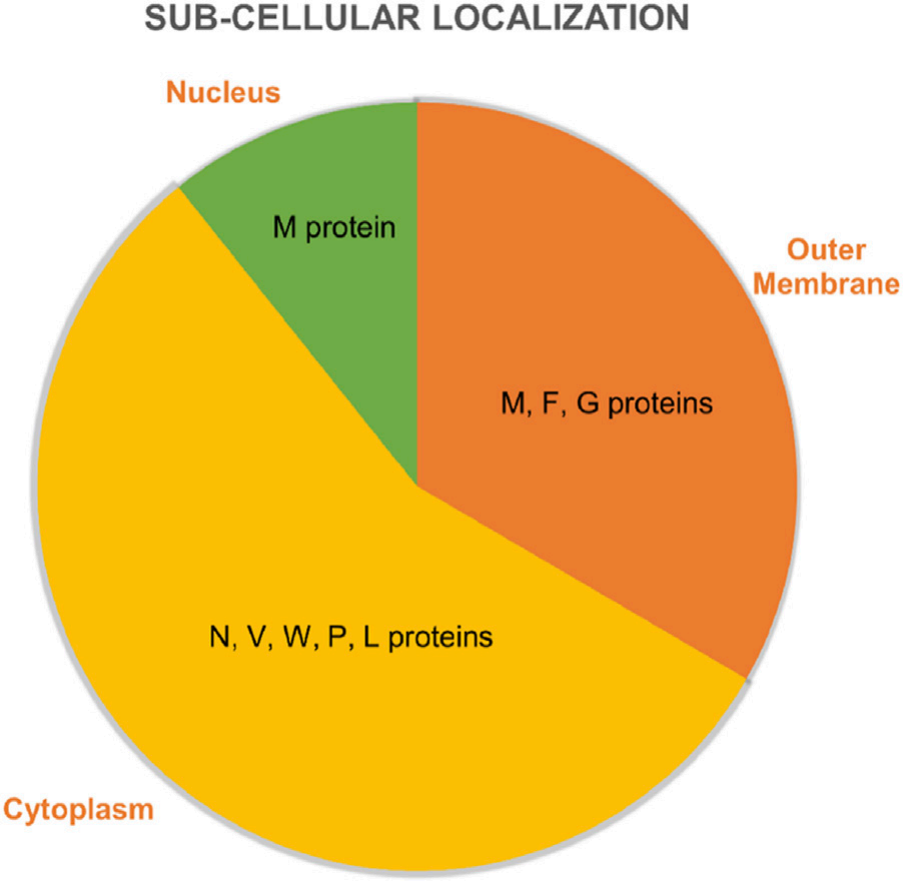
Subcellular localization and distribution of structural and functional proteins into different compartments of NiV.

The sub-cellular localization of NiV core proteins showed that out of the five core proteins, three (F, M, and G) were extracellular proteins (Figure 4). To choose the effective antigenic protein, antigenicity was also computed, although two of the proteins had antigenicities above the 0.45 threshold. Only the F (Fusion Glycoprotein) and the G (Attachment Glycoprotein) protein had significant antigenicity.

### 3.4 Predicting T-cell epitopes

The epitopes of 9-mer amino acid residues of F and G proteins for MHC-I and II was studied to target the most alleles in human populations. Multi-allelic CD4^+^ and CD8^+^ T-cell epitopes of antigenic proteins (F and G) of NiV were predicted (Table 3).

**TABLE 3:** Potential CD4^+^ and CD8^+^ T-cell epitopes of antigenic proteins (F and G) of NiV.

Classes	NiV proteins	Epitopes	No. of alleles	Position	Antigenicity	Immunogenicity	Allergenicity	Molecular weight (KDa)
MHC Class-I (CD4^+^)	F Protein	VNYNSEGIA	5	436–444	0.8164	0.0318	+	9.66
		PNFILVRNT	4	312–320	0.8719	0.23988	+	10.73
		IKMIPNVSN	4	59–67	0.6047	−0.00179	+	10.15
	G Protein	GKYDKVMPY	4	343–351	0.6545	−0.3047	+	11
		ILKPKLISY	5	197–206	0.8331	−0.28594	+	10.74
		KNKIWCISL	5	569–578	2.3553	0.24453	+	11.04
MHC Class-II (CD8^+^)	F Protein	VILNKRYYS	20	11-Mar	1.1435	0.1915	+	11.55
		ILVRNTLIS	19	316–324	0.7085	0.23351	+	10.28
		VKLQETAEK	10	159–167	0.7777	0.18541	+	10.07
	G Protein	LRNIEKGKY	14	337–346	1.2281	0.22265	+	11.2
		FLIDRINWI	11	512–521	0.7847	0.31092	+	11.89
		FLLKNKIWC	16	566–574	1.765	0.25014	+	11.64

### 3.5 Physico-chemical parameters of epitopes

The immunogenicity prediction of F and G protein for MHC class I revealed that the epitopes are highly immunogenic. The molecular weight of the F and G proteins is more than 60 kDa highlighting the significant antigenicity. The solubility data demonstrated that these epitopes are soluble at pH 7, as the positive value over 0 indicates high solubility. All epitopes were low-level allergens, according to the allergenicity analysis. Therefore, the three MHC-I epitopes (PNFILVRNT, VNYNSEGIA, and IKMIPNVSN) and the three MHC-II epitopes (ILVRNTLIS, VKLQETAEK, and VILNKRYYS) of the F proteins, and the three MHC-I (ILKPKLISY, KNKIWCISL, and GKYDKVMPY) and three MHC-II (FLLKNKIWC, LRNIEKGKY, and FLIDRINWI) epitopes of the G protein were selected as potential epitopes based on the physicochemical properties. Each epitope is non-toxic based on the significant SVM threshold value of 0.5. The substantial SVM indicates a negative value. The half-life is 1.1 h indicating a lower range and 100 h as the highest range demonstrating best for epitopes. These epitopes are stable peptides based on the threshold of stability index (< 40). The aliphatic index and theoretical pI values showed the significant score (Table 4).

**TABLE 4:** Physico-chemical properties of MHC-I and II T-cell epitopes of antigenic proteins (F and G protein) of NiV.

Classes	NiV proteins	Epitopes	Binding affinity	Toxicity	Solubility	Hydropathicity (GRAVY)	SVM score	Charge	Half-life (hours)	Theoretical pI	Instability index	Aliphatic index
MHC Class-I (CD4^+^)	F Proteins	VNYNSEGIA	−10.1561	Non-Toxic	1.726011	−0.28	−0.77	−1	100	4	20.86	86.67
		PNFILVRNT	−9.1994	Non-Toxic	1.028095	−1.04	−1.04	1	>20	10.18	17.87	118.89
		IKMIPNVSN	−9.3395	Non-Toxic	1.675904	0.2	−0.69	1	20	8.75	32.48	118.89
	G protein	GKYDKVMPY	−10.7214	Non-Toxic	1.637499	−1.09	−0.85	1	30	8.43	55.72	32.22
		ILKPKLISY	−9.4375	Non-Toxic	1.467253	0.57	−1.42	2	20	9.7	3	173.33
		KNKIWCISL	−9.2548	Non-Toxic	0.444427	0.26	−0.03	2	1.3	9.31	25.77	130
MHC Class-II (CD8^+^)	F Proteins	VILNKRYYS	−12.5699	Non-Toxic	1.489251	−0.31	−0.33	2	100	9.7	98.16	118.89
		ILVRNTLIS	−11.103	Non-Toxic	1.201023	1.26	−1.12	1	20	9.75	34.57	205.56
		VKLQETAEK	−11.526	Non-Toxic	2.110302	0.3	−0.64	0	100	6.11	53.88	86.67
	G protein	LRNIEKGKY	−11.5854	Non-Toxic	2.166611	−1.41	−0.94	2	5.5	9.7	122.78	86.67
		FLIDRINWI	−11.2844	Non-Toxic	1.04248	0.86	−0.8	0	1.1	5.84	−11.01	173.33
		FLLKNKIWC	−11.1999	Non-Toxic	1.306684	0.58	−0.24	2	1.1	9.31	16.33	130

### 3.6 Conservational, proteasomal cleavage and quality assessement

**FIGURE 5:**
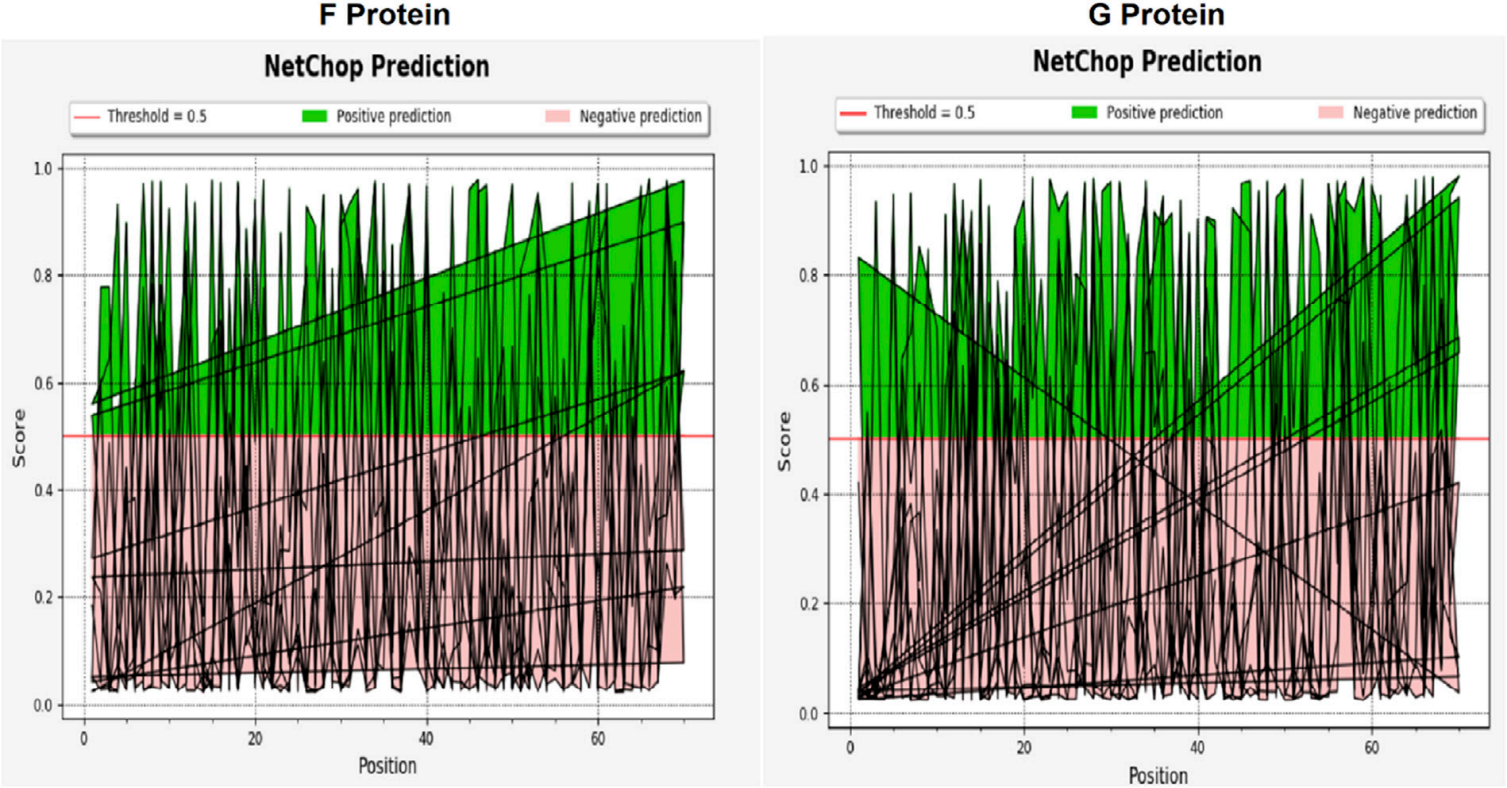
Proteasomal cleavage analysis of F and G proteins of NiV isolates.

**FIGURE 6:**
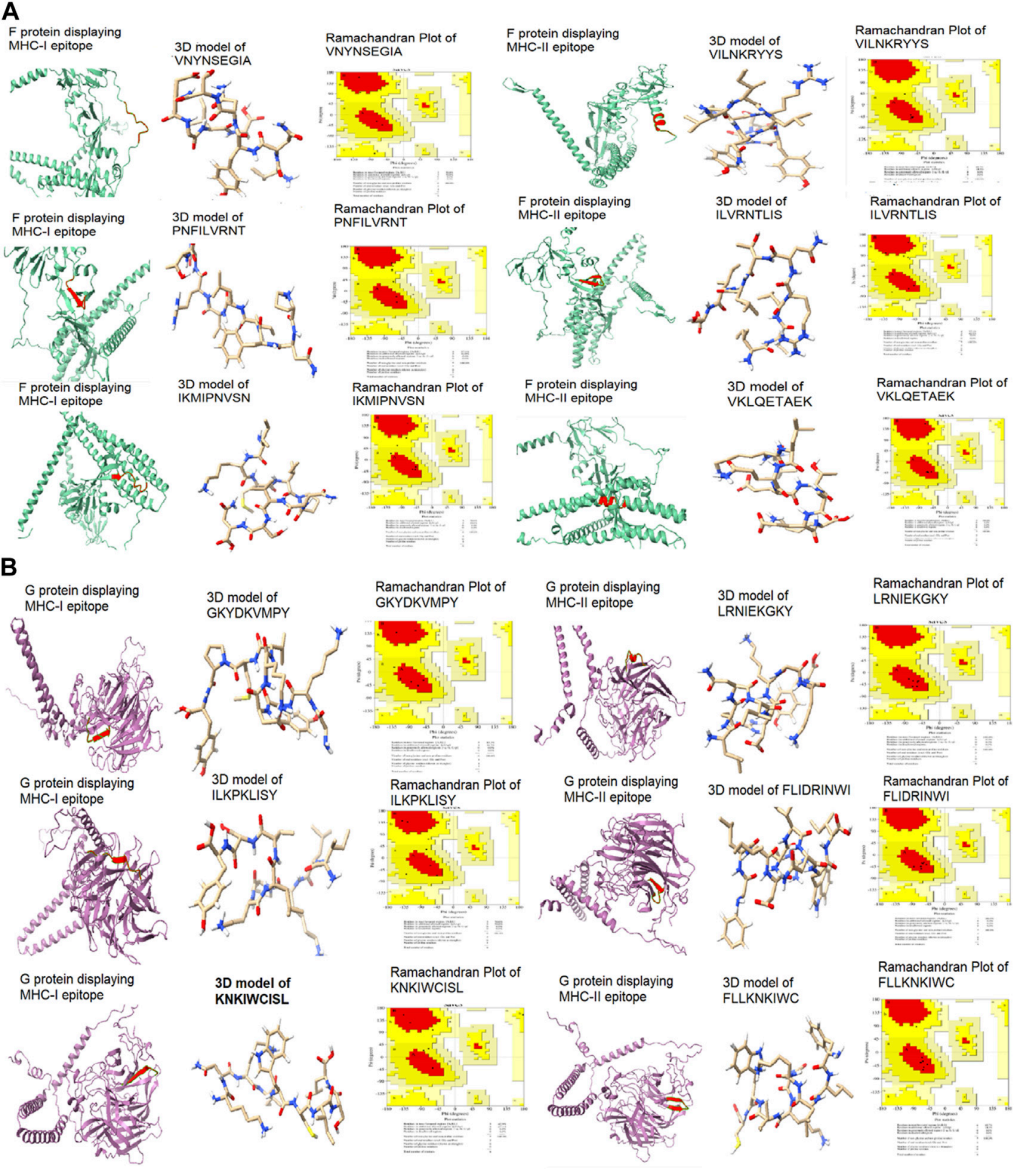
3D models of potential **e**pitopes of MHC class-I and II validated by Ramachandran plots **(A)** F protein **(B)** G protein.

All epitopes are conserved indicating more than 70% conservation. The population coverage analysis showed that these epitopes are 17–51 percent conserved in entire population against multiple alleles. To determine the number of cleavage sites, the proteasomal cleavage of F and G protein showed a significant score greater than 0.5 indicating an amino acid’s ability to cleave at a favorable position (Figure 5). The quality of 3D models of epitope is assessed by QMEAN score (Table 5). Each epitope with a QMEAN score less than −4.0 indicates the quality of models. The Ramachandran plots also verified the quality of the models indicating 80% of amino acids residues are in favourable region (Figure 6).

**TABLE 5:** Quality assessment, conservational, and population coverage analysis of NiV epitopes.

Proteins	Classes	Epitopes	QMEAN score	Conservational analysis (%)	% of population coverage
F Protein	MHC-I	VNYNSEGIA	−1.56	97.56	31.97
		PNFILVRNT	−4.42	100.00	31.97
		IKMIPNVSN	−5.61	100.00	33.88
	MHC-II	VILNKRYYS	−0.48	92.68	45.74
		ILVRNTLIS	−4.35	100.00	31.94
		VKLQETAEK	−1.51	100.00	19.15
G Protein	MHC-I	GKYDKVMPY	−1.32	78.04	37.25
		ILKPKLISY	2.02	100.00	17.71
		KNKIWCISL	−0.65	100.00	20.69
	MHC-II	LRNIEKGKY	−0.25	78.04	36.06
		FLIDRINWI	−4.01	100.00	28.05
		FLLKNKIWC	−0.51	100.00	23.74

### 3.7 Virus to host protein-protein interaction

**FIGURE 7:**
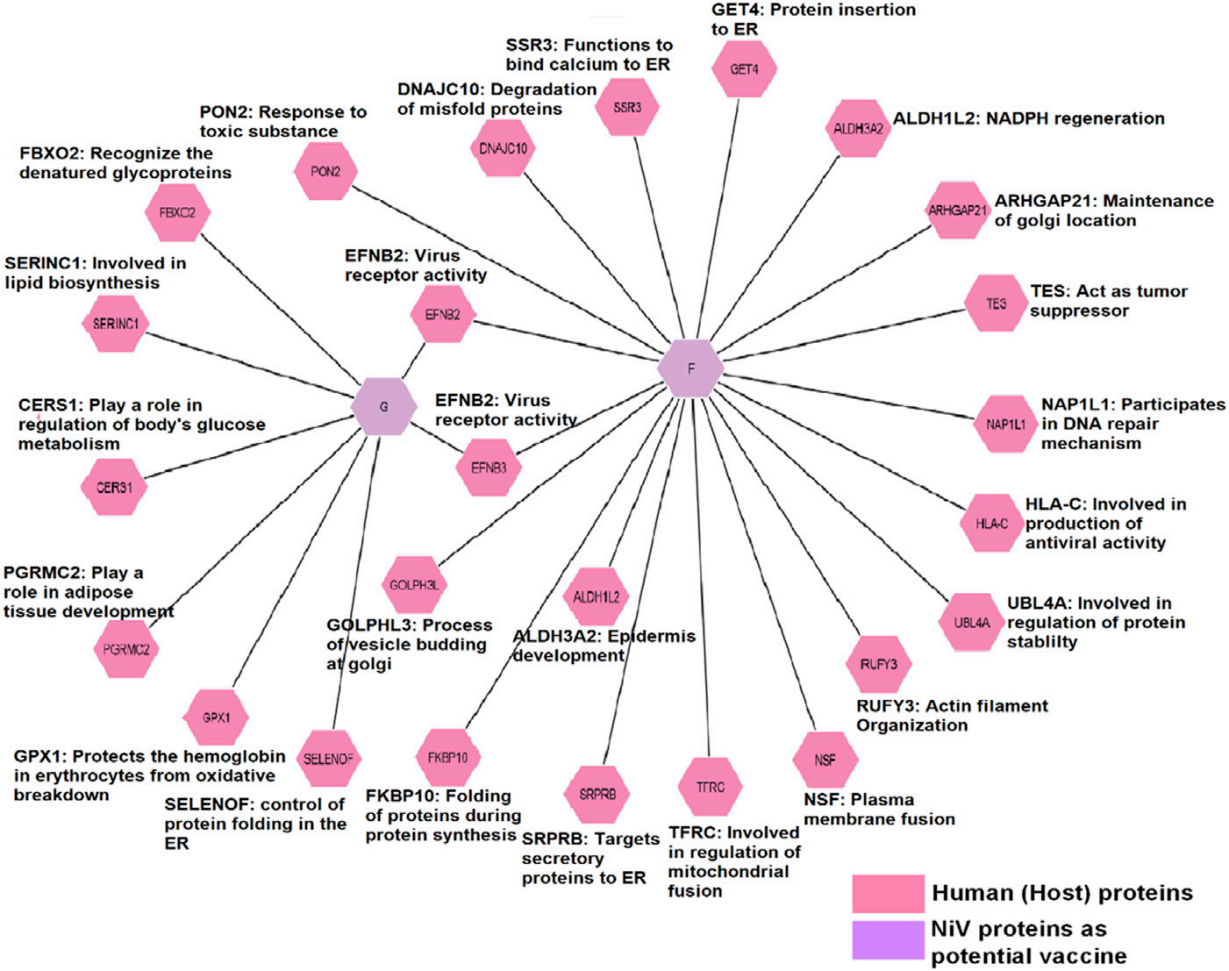
Interactomic analysis of viral proteins with host proteins indicating the transformational modifications and pathogenesis. In a network, nodes indicate proteins while edges show interactions visualized by different colors.

The two NiV proteins G and F perform the activities of attachment and fusion during infection of the host, but they also interact with many other human proteins. Both the protein G and F interact with EFNB3 and EFNB2, which are crucial for viral entry. SELENOF, FBXO2, GPX1, SERINC1, CERS1, and PGRMC2 all interact with the G protein. FKBP10, RUFY3, DNAJC10, GET4, ALDH1L2, ARHGAP21, ALDH3A2, PON2, TES, NSF, UBL 4A, HLA-C, TFRC, and NAP1L1 are interacting with F protein. The functional interaction of these human and NiV proteins are responsible for pathogenesis (Figure 7).

### 3.8 Binding energy and molecular dynamics

**FIGURE 8:**
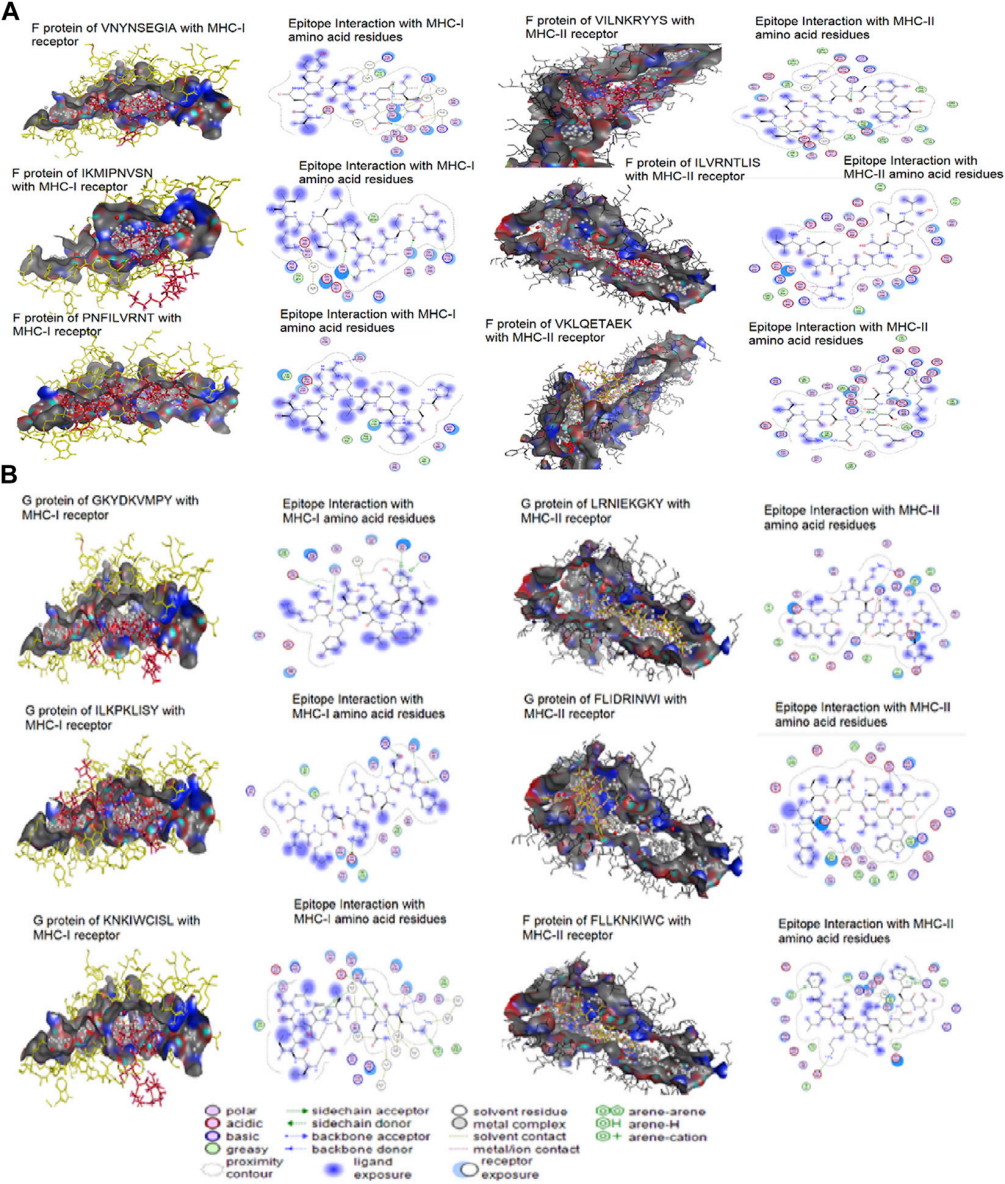
Molecular docking of potential epitopes models as ligands with MHC class I and II molecules as targets **(A)** F protein **(B)** G protein.

The epitopes were docked with MHC-I and II molecules. The most crucial screening criterion is the binding energies of epitopes. The lowest binding affinity value demonstrates a strong interaction with the target. Each MHC-I and II epitope displayed the lowest score of binding energy. The MHC-I epitopes PNFILVRNT, VNYNSEGIA, and IKMIPNVSN of the F protein and ILKPKLISY, GKYDKVMPY, and KNKIWCISL of the G protein, showed binding affinities of −9.19, −10.15, −9.33, −9.43, −10.72, and −9.25 kcal/mol respectively. These epitopes demonstrated interactions with the MHC-I amino acids histidine, isoleucine, alanine, tyrosine, phenylalanine, lysine, valine, arginine, glutamic acid, glycine, asparagine, serine, glutamine, and tryptophan. The MHC-II epitopes ILVRNTLIS, VILNKRYYS, and VKLQETAEK of the F protein and FLIDRINWI, LRNIEKGKY, and FLLKNKIWC of the G protein have binding affinities of −11.10, −12.56, −11.52, −11.28, −11.58, and −11.19 kcal/mol. The amino acids glutamic acid, glycine, phenylalanine, asparagine, leucine, glutamine, arginine, lysine, proline, histidine, threonine, serine, vaine, and isoleucine of MHC-II were discovered to interact with these epitopes (Figure 8).

**FIGURE 9:**
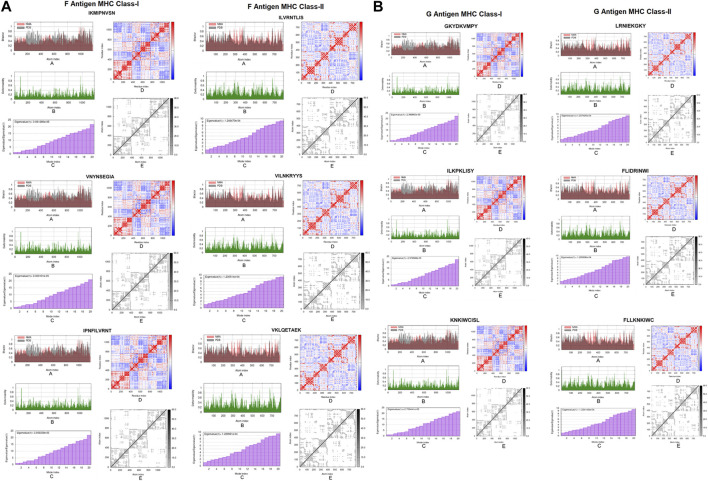
Molecular dynamics of epitope-MHC complex; Each complex’stability was assessed through (a) B-factor, (b) deformability, (c) eigenvalue, (d) covariance index and (e) elastic network model. **(A)** F protein **(B)** G protein.

Molecular dynamics simulation was performed to assess the molecular behavior and stability of each docked complex. The complex’s deformability relied on the individual distortion of each residue, represented by the hinges in the chain. The MHC-I epitopes PNFILVRNT, VNYNSEGIA, and IKMIPNVSN of the F protein and ILKPKLISY, GKYDKVMPY, and KNKIWCISL of the G protein showed the eigen values of 3.05e^−05^, 3.00e^−05^, 3.06e^−05^, 2.97e^−05^, 2.98e^−05^ and 2.70e^−05^ respectively. The MHC-II epitopes ILVRNTLIS, VILNKRYYS, and VKLQETAEK of the F protein and FLIDRINWI, LRNIEKGKY, and FLLKNKIWC of the G protein displayed the eigen values of 1.24e^−04^, 1.23e^−04^, 1.22e^−04^, 1.22e^−04^, 1.234e^−04^ and 1.237e^−04^ respectively. The B-factor scores were equivalent to RMS. The coupling between pairs of residues was explained by the covariance matrix, where various pairs displayed correlated, anti-correlated, or uncorrelated motions, which were represented by red, blue, and white colors, respectively. The elastic network model displayed the pair of atoms connected by springs in accordance with the degree of stiffness between them. This stiffness was represented by color, moving from lighter grey with softer strings to darker grey with stiffer strings (Figure 9).

### 3.9 Codon optimization and cloning

**FIGURE 10:**
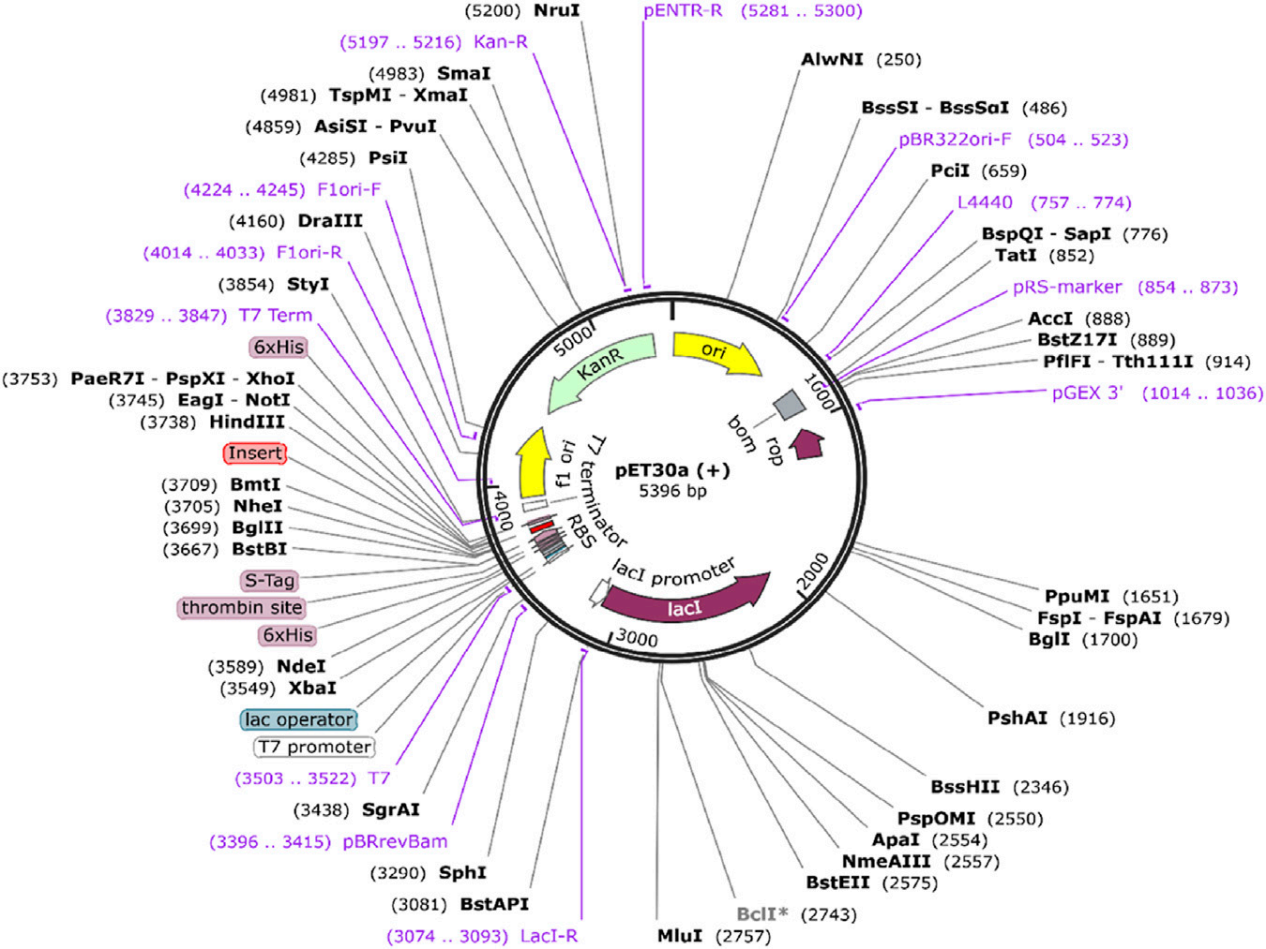
*In silico* cloned codon-optimized *epitopes* into *E. coli* strain K12 expression system. The inserted construct DNA sequence is shown in red color.

The epitopes expression in the K12 strain of *E. coli* was verified using *in silico* cloning. Each codon-optimized epitope has a 39 bp sequence and was created using the JCAT server. The GC content ranges between 30% and 70%, while the CAI value barrier is between 0.8 and 1.0. For both MHC class I and II epitopes of F and G proteins, every optimized epitope had a CAI value of 1.0 and a GC concentration of more than 37%. All the epitopes were restricted using the NheI and HindIII enzymes, and high expression levels of the epitopes were observed at several cloning regions in pET30a (+) (Figure 10).

## 4 Discussion

Reverse vaccinology is time and cost effective, as it benefits from breakthroughs in genomics and proteomics, whereas traditional approaches are expensive and time-consuming (Nezafat et al., 2016). Several vaccines designed through *in silico* techniques have passed successful clinical testing. The COVID-19 vaccine was designed with a lot of aid from this innovative procedures (Oli and Rowaiye, 2022). Nipah virus is a fatal infection that may spread from person to person, from animal to animal, and from excretory materials of bats to humans. It causes encephalitis and respiratory collapse in humans (Singh et al., 2019). Because of its high death rate, the zoonotic potential of human-to-human transmission, and the lack of a vaccine, NiV is observed as a global health concern by the World Health Organization (WHO), National Institute of Allergy and Infectious Diseases (NIAID), Research and Development activities (R&D), and Centre for Disease Control and Prevention (CDC). The most recent incidence proved that the virus first appeared in India in September 2021 (Skowron et al., 2021).

Currently, the WHO has identified a number of cases of NiV, but yet there is no vaccination. Till now, 721 cases of NiV with 419 deaths were reported in India, Bangladesh, Malaysia and Singapore (Chattu et al., 2018; WHO, 2023a; Nazmunnahar et al., 2023). Recently, a 6th outbreak of NiV occurred in India since 2001. Six NiV cases including two deaths were reported in Kerala, India between 12 and 15 September (WHO, 2023b). Despite the high fatality rates associated with Nipah outbreaks (>70% in Southeast Asia), there are currently no approved medications or vaccines. To avoid uncontrollable situation, it is necessary to develop therapeutic strategy or suitable vaccine to treat NiV (Sen et al., 2019).

To design epitope based vaccines of NiV, the complete proteomic sequence information is available now. Previous studies predicted B and T-cell epitopes of F, V, W, and G proteins, however they missed important subcellular localization and molecular weight estimation (Kamthania and Sharma, 2015; Soltan et al., 2021). Some studies predicted the epitopes of F protein leaving the other antigenic proteins (Ali et al., 2015). For immunoinformatics based vaccine designing, the population coverage analysis is vital that reflects the broader spectrum of epitopes (Moten et al., 2022).

In current study, we applied an integrative system-level immunoinformatics based framework to predict the CD4^+^ and CD8^+^ T-cell epitopes of NiV involving rigorous analysis of antigenicity predictions, screening, protein to protein interactions, and *in silico* binding affinity analysis. Pangenomic to proteomic level analysis showed that the NiV has nine structural and functional proteins, each involved in pathogenesis. The subcellular localization analysis is important as potential vaccine candidates are either membrane bound or extracellular proteins and we observed that among five, three proteins (F, M, and G) are surface and the remaining are intracellular proteins.

F and G are significant proteins with molecular weight of more than 60 kDa. T-cell epitopes of G and F proteins are antigenic, as evidenced by the primary screening of the epitopes based on substantial cut off parameters. We observed the binding affinity of these peptides with MHCs molecules to analyze the pharmacological activities and the immunological response (Moten et al., 2022). All epitopes showed strong physical and chemical characteristics and these criteria demonstrated their capacity to elicit an immune response.

The pan-genome analysis is important to find and provide information for efficient findings on core and accessory genomes of pathogenic microbes. The core proteins could be used for epitopes predictions as conserved antigenic proteins are potential vaccine candidates.

## 5 Conclusion

In conclusion, we found 12 antigenic T-cell epitopes of F and G proteins of NiV, as they have potential affinity for HLA alleles. Two epitopes of G protein including KNKIWCISL of MHC-I, and FLLKNKIWC of MHC-II, and similarly two epitopes of F protein including PNFILVRNT of class MHC-I and ILNKRYYS of MHC-II showed high antigenicity values of 2.35, 1.76, 0.87 and 1.14 kcal/mol respectively. The immunoinformatics based approach for designing NiV vaccine is an important step to cope against this infection.

## Data Availability

The original contributions presented in the study are included in the article/Supplementary Material, further inquiries can be directed to the corresponding authors.

## References

[B1] Adams H.-P. Koziol J. A. (1995). Prediction of binding to MHC class I molecules. J. Immunol. Methods. 185 (2), 181–190. 10.1016/0022-1759(95)00111-M 7561128

[B2] Agrawal R. Murmu J. Pattnaik S. Kanungo S. Pati S. (2023). Bangladesh sees spike in Nipah virus cases: a matter of public health concern? New Microbes New Infect. 53, 101119. 10.1016/j.nmni.2023.101119 37090952 PMC10119947

[B3] Ali M. T. Morshed M. M. Hassan F. (2015). A computational approach for designing a universal epitope-based peptide vaccine against Nipah virus. Interdiscip. Sci. 7 (2), 177–185. 10.1007/s12539-015-0023-0 26156209

[B4] Aljofan M. (2013). Hendra and Nipah infection: emerging paramyxoviruses. Virus Res. 177 (2), 119–126. 10.1016/j.virusres.2013.08.002 23954578

[B5] Ambat A. S. Zubair S. M. Prasad N. Pundir P. Rajwar E. Patil D. S. (2019). Nipah virus: a review on epidemiological characteristics and outbreaks to inform public health decision making. J. Infect. Public. 12 (5), 634–639. 10.1016/j.jiph.2019.02.013 30808593

[B6] Ang B. S. Lim T. C. Wang L. (2018). Nipah virus infection. J. Clin. Microbiol. 56 (6), e01875–e01817. 10.1128/JCM.01875-17 29643201 PMC5971524

[B7] Bui H.-H. Sidney J. Dinh K. Southwood S. Newman M. J. Sette A. (2006). Predicting population coverage of T-cell epitope-based diagnostics and vaccines. BMC Bioinform 7 (1), 153–155. 10.1186/1471-2105-7-153 PMC1513259 16545123

[B8] Bui H.-H. Sidney J. Li W. Fusseder N. Sette A. (2007). Development of an epitope conservancy analysis tool to facilitate the design of epitope-based diagnostics and vaccines. BMC Bioinform 8 (1), 361–366. 10.1186/1471-2105-8-361 PMC2233646 17897458

[B9] Calis J. J. Maybeno M. Greenbaum J. A. Weiskopf D. De Silva A. D. Sette A. (2013). Properties of MHC class I presented peptides that enhance immunogenicity. PloS Comp. Biol. 9 (10), e1003266. 10.1371/journal.pcbi.1003266 PMC3808449 24204222

[B10] Chatr-Aryamontri A. Ceol A. Palazzi L. M. Nardelli G. Schneider M. V. Castagnoli L. (2007). MINT: the Molecular INTeraction database. Nucleic Acids Res. 35 (1), D572–D574. 10.1093/nar/gkl950 17135203 PMC1751541

[B11] Chattu V. K. Kumar R. Kumary S. Kajal F. David J. K. (2018). Nipah virus epidemic in southern India and emphasizing “One Health” approach to ensure global health security. J. Fam. Med. Primar. care. 7 (2), 275–283. 10.4103/jfmpc.jfmpc_137_18 PMC6060941 30090764

[B12] Colovos C. Yeates T. O. (1993). Verification of protein structures: patterns of nonbonded atomic interactions. Protein Sci. 2 (9), 1511–1519. 10.1002/pro.5560020916 8401235 PMC2142462

[B13] Cox R. M. Plemper R. K. (2017). Structure and organization of paramyxovirus particles. Curr. Opin. Virol. 24, 105–114. 10.1016/j.coviro.2017.05.004 28601688 PMC5529233

[B14] Dimitrov I. Naneva L. Doytchinova I. Bangov I. (2014). AllergenFP: allergenicity prediction by descriptor fingerprints. J. Bioinform. 30 (6), 846–851. 10.1093/bioinformatics/btt619 24167156

[B15] Doytchinova I. A. Flower D. R. (2007). VaxiJen: a server for prediction of protective antigens, tumour antigens and subunit vaccines. BMC Bioinform 8 (1), 4–7. 10.1186/1471-2105-8-4 PMC1780059 17207271

[B16] Du Z. Su H. Wang W. Ye L. Wei H. Peng Z. (2021). The trRosetta server for fast and accurate protein structure prediction. Nat. Protoc. 16 (12), 5634–5651. 10.1038/s41596-021-00628-9 34759384

[B17] Edgar R. C. Batzoglou S. (2006). Multiple sequence alignment. COSB 16 (3), 368–373. 10.1016/j.sbi.2006.04.004 16679011

[B18] Gasteiger E. Hoogland C. Gattiker A. Wilkins M. R. Appel R. D. Bairoch A. (2005). Protein identification and analysis tools on the ExPASy server. J. Proteomics 112, 531–552. 10.1385/1-59259-890-0:571 10027275

[B19] Goddard T. D. Huang C. C. Meng E. C. Pettersen E. F. Couch G. S. Morris J. H. (2018). UCSF ChimeraX: meeting modern challenges in visualization and analysis. Protein Sci. 27 (1), 14–25. 10.1002/pro.3235 28710774 PMC5734306

[B20] Gopalakrishnan K. Sowmiya G. Sheik S. Sekar K. (2007). Ramachandran plot on the web (2.0). Protein Pept. 14 (7), 669–671. 10.2174/092986607781483912 17897092

[B21] Grote A. Hiller K. Scheer M. Münch R. Nörtemann B. Hempel D. C. (2005). JCat: a novel tool to adapt codon usage of a target gene to its potential expression host. Nucleic Acids Res. 33 (2), W526–W531. 10.1093/nar/gki376 15980527 PMC1160137

[B22] Guedes I. A. Pereira F. S. Dardenne L. E. (2018). Empirical scoring functions for structure-based virtual screening: applications, critical aspects, and challenges. Front. Pharmacol. 9, 1089. 10.3389/fphar.2018.01089 30319422 PMC6165880

[B23] Gupta S. Kapoor P. Chaudhary K. Gautam A. Kumar R. Consortium O. S. D. D. (2013). *In silico* approach for predicting toxicity of peptides and proteins. PloS one 8 (9), e73957. 10.1371/journal.pone.0073957 24058508 PMC3772798

[B24] Harcourt B. H. Lowe L. Tamin A. Liu X. Bankamp B. Bowden N. (2005). Genetic characterization of Nipah virus, Bangladesh, 2004. Emerg. Infect. Dis. 11 (10), 1594–1597. 10.3201/eid1110.050513 16318702 PMC3366751

[B25] Hassan A. Naz A. Obaid A. Paracha R. Z. Naz K. Awan F. M. (2016). Pangenome and immuno-proteomics analysis of Acinetobacter baumannii strains revealed the core peptide vaccine targets. BMC genomics 17 (1), 732–825. 10.1186/s12864-016-2951-4 27634541 PMC5025611

[B26] Hauser N. Gushiken A. C. Narayanan S. Kottilil S. Chua J. V. (2021). Evolution of Nipah virus infection: past, present, and future considerations. Trop. Med. Infect. Dis. 6 (1), 24. 10.3390/tropicalmed6010024 33672796 PMC8005932

[B27] Jalili V. Afgan E. Gu Q. Clements D. Blankenberg D. Goecks J. (2020). The Galaxy platform for accessible, reproducible and collaborative biomedical analyses: 2020 update. Nucleic Acids Res. 48 (W1), W395–W402. 10.1093/nar/gkaa434 32479607 PMC7319590

[B28] Kamthania M. Sharma D. (2015). Screening and structure-based modeling of T-cell epitopes of Nipah virus proteome: an immunoinformatic approach for designing peptide-based vaccine. 3 Biotech. 5 (6), 877–882. 10.1007/s13205-015-0303-8 PMC4624138 28324411

[B29] King A. M. Lefkowitz E. Adams M. J. Carstens E. B. (2011). Virus taxonomy: ninth report of the international committee on taxonomy of viruses. Netherlands: Elsevier.

[B30] Moten D. Kolchakova D. Todorov K. Mladenova T. Dzhambazov B. (2022). Design of an epitope-based peptide vaccine against the major allergen amb a 11 using immunoinformatic approaches. Protein J. 41, 315–326. 10.1007/s10930-022-10050-z 35362839 PMC8972712

[B31] Nazmunnahar A. Ahmed I. Roknuzzaman A. Islam M. R. (2023). The recent Nipah virus outbreak in Bangladesh could be a threat for global public health: a brief report. Health Scien Repor 6 (7), e1423. 10.1002/hsr2.1423 PMC10336337 37448729

[B32] Nezafat N. Karimi Z. Eslami M. Mohkam M. Zandian S. Ghasemi Y. (2016). Designing an efficient multi-epitope peptide vaccine against *Vibrio cholerae* via combined immunoinformatics and protein interaction based approaches. Comput. Biol. Chem. 62, 82–95. 10.1016/j.compbiolchem.2016.04.006 27107181

[B33] Nielsen M. Lundegaard C. Lund O. Keşmir C. (2005). The role of the proteasome in generating cytotoxic T-cell epitopes: insights obtained from improved predictions of proteasomal cleavage. Immunogenetics 57 (1), 33–41. 10.1007/s00251-005-0781-7 15744535

[B34] Ochoa R. Laio A. Cossio P. (2019). Predicting the affinity of peptides to major histocompatibility complex class II by scoring molecular dynamics simulations. J. Chem. Inf. Model. 59 (8), 3464–3473. 10.1021/acs.jcim.9b00403 31290667

[B35] Ochoa R. Lunardelli V. A. S. Rosa D. S. Laio A. Cossio P. (2022). Multiple-allele MHC class II epitope engineering by a molecular dynamics-based evolution protocol. Front. Immunol. 13, 862851. 10.3389/fimmu.2022.862851 35572587 PMC9094701

[B36] Okumura H. Itoh S. G. Okamoto Y. (2007). Explicit symplectic integrators of molecular dynamics algorithms for rigid-body molecules in the canonical, isobaric-isothermal, and related ensembles. J. Chem. Phys. 126 (8), 084103. 10.1063/1.2434972 17343436

[B37] Oli A. N. Obialor W. O. Ifeanyichukwu M. O. Odimegwu D. C. Okoyeh J. N. Emechebe G. O. (2020). Immunoinformatics and vaccine development: an overview. Immunotargets Ther. 9, 13–30. 10.2147/ITT.S241064 32161726 PMC7049754

[B38] Oli A. N. Rowaiye A. B. (2022). “Vaccine types and reverse vaccinology,” in Vaccinology and methods in vaccine research (Netherlands: Elsevier), 31–55. 10.1016/B978-0-323-91146-7.00013-5

[B39] Page A. J. Cummins C. A. Hunt M. Wong V. K. Reuter S. Holden M. T. (2015). Roary: rapid large-scale prokaryote pan genome analysis. J. Bioinform. 31 (22), 3691–3693. 10.1093/bioinformatics/btv421 PMC4817141 26198102

[B40] Pickett B. E. Sadat E. L. Zhang Y. Noronha J. M. Squires R. B. Hunt V. (2012). ViPR: an open bioinformatics database and analysis resource for virology research. Nucleic Acids Res. 40 (D1), D593–D598. 10.1093/nar/gkr859 22006842 PMC3245011

[B41] Sen N. Kanitkar T. R. Roy A. A. Soni N. Amritkar K. Supekar S. (2019). Predicting and designing therapeutics against the Nipah virus. PLoS Negl. Trop. Dis. 13 (12), e0007419. 10.1371/journal.pntd.0007419 31830030 PMC6907750

[B42] Shannon P. Markiel A. Ozier O. Baliga N. S. Wang J. T. Ramage D. (2003). Cytoscape: a software environment for integrated models of biomolecular interaction networks. Genome Res. 13 (11), 2498–2504. 10.1101/gr.1239303 14597658 PMC403769

[B43] Shariff M. (2019). Nipah virus infection: a review. Epidemiol. Infect. 147, e95. 10.1017/S0950268819000086 30869046 PMC6518547

[B44] Sharma V. Kaushik S. Kumar R. Yadav J. P. Kaushik S. (2019). Emerging trends of Nipah virus: a review. Rev. Med. virology (ISO4). 29 (1), e2010. 10.1002/rmv.2010 PMC7169151 30251294

[B45] Shen H.-B. Chou K.-C. (2010). Virus-mPLoc: a fusion classifier for viral protein subcellular location prediction by incorporating multiple sites. J. Biomol. Struct. 28 (2), 175–186. 10.1080/07391102.2010.10507351 20645651

[B46] Shen Y. Maupetit J. Derreumaux P. Tufféry P. (2014). Improved PEP-FOLD approach for peptide and miniprotein structure prediction. J. Chem. Theory Comput. 10 (10), 4745–4758. 10.1021/ct500592m 26588162

[B47] Sherman R. M. Salzberg S. L. (2020). Pan-genomics in the human genome era. Nat. Rev. Genet. 21 (4), 243–254. 10.1038/s41576-020-0210-7 32034321 PMC7752153

[B48] Singh H. Raghava G. (2001). ProPred: prediction of HLA-DR binding sites. J. Bioinform. 17 (12), 1236–1237. 10.1093/bioinformatics/17.12.1236 11751237

[B49] Singh R. K. Dhama K. Chakraborty S. Tiwari R. Natesan S. Khandia R. (2019). Nipah virus: epidemiology, pathology, immunobiology and advances in diagnosis, vaccine designing and control strategies–a comprehensive review. Vet. Q. 39 (1), 26–55. 10.1080/01652176.2019.1580827 31006350 PMC6830995

[B50] Skowron K. Bauza-Kaszewska J. Grudlewska-Buda K. Wiktorczyk-Kapischke N. Zacharski M. Bernaciak Z. (2021). Nipah virus–another threat from the world of zoonotic viruses. Front. Microbiol. 12, 811157. 10.3389/fmicb.2021.811157 35145498 PMC8821941

[B51] Soltan M. A. Eldeen M. A. Elbassiouny N. Mohamed I. El-Damasy D. A. Fayad E. (2021). Proteome based approach defines candidates for designing a multitope vaccine against the Nipah virus. Int. J. Mol. Sci. 22 (17), 9330. 10.3390/ijms22179330 34502239 PMC8431361

[B52] Soman Pillai V. Krishna G. Valiya Veettil M. (2020). Nipah virus: past outbreaks and future containment. Viruses 12 (4), 465. 10.3390/v12040465 32325930 PMC7232522

[B53] Sormanni P. Amery L. Ekizoglou S. Vendruscolo M. Popovic B. (2017). Rapid and accurate *in silico* solubility screening of a monoclonal antibody library. Sci. Rep. 7 (1), 8200–8209. 10.1038/s41598-017-07800-w 28811609 PMC5558012

[B54] Studer G. Rempfer C. Waterhouse A. M. Gumienny R. Haas J. Schwede T. (2020). QMEANDisCo—distance constraints applied on model quality estimation. Bioinformatics 36 (6), 1765–1771. 10.1093/bioinformatics/btz828 31697312 PMC7075525

[B55] Sun B. Jia L. Liang B. Chen Q. Liu D. (2018). Phylogeography, transmission, and viral proteins of Nipah virus. Virol. Sin. 33 (5), 385–393. 10.1007/s12250-018-0050-1 30311101 PMC6235768

[B56] Tomar N. Singh V. Marla S. Chandra R. Kumar R. Kumar A. (2010). Molecular docking studies with rabies virus glycoprotein to design viral therapeutics. Indian J. Pharm. Sci. 72 (4), 486–490. 10.4103/0250-474X.73905 21218060 PMC3013581

[B57] WHO (2023a). Nipah virus infection - Bangladesh. Available at: https://www.who.int/emergencies/disease-outbreak-news/item/2023-DON442 (Accessed October 24, 2023).

[B58] WHO (2023b). Nipah virus infection - India. Available at: https://www.who.int/emergencies/disease-outbreak-news/item/2023-DON490 (Accessed October 23, 2023).

[B59] Yahya E. B. Alqadhi A. M. Abdulsamad M. A. Allaq A. A. (2021). Asian Nipah virus and the potential of new pandemic. PJBT 18 (1-2), 17–22. 10.34016/pjbt.2021.18.1.17

